# Supplementary material for: eIF1A residues implicated in cancer stabilize translation preinitiation complexes and favor suboptimal initiation sites in yeast
Source: eLife. 2017 Dec 5;6:e31250. doi: 10.7554/eLife.31250 (PMC5756025; doi:10.7554/eLife.31250)
Supplement: Supplementary file 1. — Table S1: Oligonucleotide primers employed for TIF11 mutagenesis in this study Table S2: Ribosome profiling datasets used for uORF identification [file elife-31250-supp1.pdf]

Supplemental Material

for

eIF1A residues implicated in cancer stabilize translation preinitiation complexes and favor suboptimal initiation sites in yeast

Supplementary Tables

Table S1. Primers used for *TIF11* mutagenesis (with mutated nucleotides underlined).

Primer	Sequence (5' to 3')	Mutation
tif11p.K3Efw	AAGTTCATCATGGGTGAGAAAAACACTAAAG	*tif11-K3E*
tif11p.K3Erev	CTTTAGTGTTTTTCTCACCCATGATGAACTT	*tif11-K3E*
tif11p.K4Dfw	TTCATCATGGGTAAGGACAACACTAAAGGTGGT	*tif11-K4D*
tif11p.K4Drev	ACCACCTTTAGTGTTGTCCTTACCCATGATGAA	*tif11-K4D*
tif11p.T6Dfw	ATGGGTAAGAAAAACGATAAAGGTGGTAAAAA	*tif11-T6D*
tif11p.T6Drev	TTTTTACCACCTTTATCGTTTTTCTTACCCAT	*tif11-T6D*
tif11p.T6Rfw	ATGGGTAAGAAAAACCGTAAAGGTGGTAAAAA	*tif11-T6R*
tif11p.T6Rrev	TTTTTACCACCTTTACGGTTTTTCTTACCCAT	*tif11-T6R*
tif11p.G8delfw	AAAAACACTAAAGGT(3ntdel)AAAAAAGGTAGAAGA	*tif11-ΔG8*
tif11p.G8delrev	TCTTCTACCTTTTTT(3ntdel)ACCTTTAGTGTTTTT	*tif11-ΔG8*
tif11p.R13Pfw	GGTGGTAAAAAAGGTCCAAGAGGTAAGAACGA	*tif11-R13P*
tif11p.R13Prev	TCGTTCTTACCTCTTGGACCTTTTTTACCACC	*tif11-R13P*
tif11p.G15Dfw	AAAAAGGTAGAAGAGATAAGAACGACTCTGA	*tif11-G15D*
tif11p.G15Drev	TCAGAGTCGTTCTTATCTCTTCTACCTTTTT	*tif11-G15D*
K7A-F	ATGGGTAAGAAAAACACTGCTGGTGGTAAAAAAGGTAGA	*tif11-K7A*
K7A-R	TCTACCTTTTTTACCACCAGCAGTGTTTTTCTTACCCAT	*tif11-K7A*
K7D-F	ATGGGTAAGAAAAACACTGATGGTGGTAAAAAAGGTAGA	*tif11- K7D*
K7D-R	TCTACCTTTTTTACCACCATCAGTGTTTTTCTTACCCAT	*tif11- K7D*
DG8G9-F	ATGGGTAAGAAAAACACTAAA(6ntdel)AAAAAAGGTAGAAG AGGT AAG	*tif11-ΔG8ΔG9*
DG8G9-R	CTTACCTCTTCTACCTTTTTT(6ntdel)TTTAGTGTTTTTCTTAC CCAT	*tif11-ΔG8ΔG9*
K10A-F	AAAAACACTAAAGGTGGTGCTAAAGGTAGAAGAGGTAAG	*tif11-K10A*

Primer	Sequence (5' to 3')	Mutation
K10A-R	CTTACCTCTTCTACCTTTAGCACCACCTTTAGTGTTTTT	*tif11-K10A*
K10D-F	AAAAACACTAAAGGTGGTGATAAAGGTAGAAGAGGTAAG	*tif11-K10D*
K10D-R	CTTACCTCTTCTACCTTTATCACCACCTTTAGTGTTTTT	*tif11-K10D*
R13A-F	AAAGGTGGTAAAAAAGGTGCTAGAGGTAAGAACGACTCT	*tif11-R13A*
R13A-R	AGAGTCGTTCTTACCTCTAGCACCTTTTTTACCACCTTT	*tif11-R13A*
R13D-F	AAAGGTGGTAAAAAAGGTGATAGAGGTAAGAA.CGACTCT	*tif11-R13D*
R13D-R	AGAGTCGTTCTTACCTCTATCACCTTTTTTACCACCTTT	*tif11-R13D*
R14A-F	GGTGGTAAAAAAGGTAGAGCTGGTAAGAACGACTCTGAC	*tif11-R14A*
R14A-R	GTCAGAGTCGTTCTTACCAGCTCTACCTTTTTTACCACC	*tif11-R14A*
R14D-F	GGTGGTAAAAAAGGTAGAGATGGTAAGAACGACTCTGAC	*tif11-R14D*
R14D-R	GTCAGAGTCGTTCTTACCATCTCTACCTTTTTTACCACC	*tif11-R14D*
K16A-F	AAAAAAGGTAGAAGAGGTGCTAACGACTCTGACGGTCCA	*tif11-K16A*
K16A-R	TGGACCGTCAGAGTCGTTAGCACCTCTTCTACCTTTTTT	*tif11-K16A*
K16D-F	AAAAAAGGTAGAAGAGGTGATAACGACTCTGACGGTCCA	*tif11-K16D*
K16D-R	TGGACCGTCAGAGTCGTTATCACCTCTTCTACCTTTTTT	*tif11-K16D*

Table S2. Ribosome profiling datasets used for uORF identification

Strain	Relevant genotype	Growth conditions	Source	GEO Accession Number
***tif11-R13P* experiment**				
PMY337/PMY338	*TIF11/SUI3*	SC, 30°C	This study	GSM2895450, GSM2895451
FZY010/FZY011	*tif11-R13P/SUI3*	SC, 30°C	This study	GSM2895498, GSM2895499
PMY335/PMY336	*TIF11/SUI3-2*	SC, 30°C	This study	GSM2895452, GSM2895453
PMY316	*tif11-R13P/ SUI3-2*	SC, 30°C	This study	GSM2895454, GSM2895455
***sui1-L96P* experiment**				
PMY30	*SUI1*	SC, 30°C	This study	GSM2895474, GSM2895475
		SC, 33°C	This study	GSM2895470, GSM2895471
PMY33	*sui1-L96P*	SC, 30°C	This study	GSM2895472, GSM2895473
		SC, 33°C	This study	GSM2895468, GSM2895469
***sui1-T15A* experiment**				
FJZ012/FJZ013	*SUI1/SUI3*	SC, 30°C	This study	GSM2895458, GSM2895459
FJZ014/FJZ015	*sui1-T15A/SUI3*	SC, 30°C	This study	GSM2895456, GSM2895457
PMY71	*SUI1/SUI3-2*	SC, 30°C	This study	GSM2895460, GSM2895461
PMY72	*sui1-T15A/SUI3-2*	SC, 30°C	This study	GSM2895462, GSM2895463
**WT experiments**				
BY4741	WT	SC, 20°C/25°C/ 30°C/36°C	This study	GSM2895476 - GSM2895491

***tif3Δ* YPD experiments**

FJZ046	WT	YPD, 23°C	This study	GSM2895466, GSM2895467
FJZ052	*tif3Δ*	YPD, 23°C	This study	GSM2895464, GSM2895465

***tif1-ts*⁻ experiment**

NSY20	*TIF1*	SC, 30°C	(Sen et al. 2015)	GSM1622004, GSM1622005
		SC, 30°C to 37°C shift	(Sen et al. 2015)	GSM1622012, GSM1622013
NSY21	*tif1-A79V*	SC, 30°C	(Sen et al. 2015)	GSM1622006, GSM1622007
		SC, 30°C to 37°C shift	(Sen et al. 2015)	GSM1622014, GSM1622015

***ded1-cs*⁻ experiment**

NSY4	*DED1*	SC, 30°C to 15°C shift	(Sen et al. 2015)	GSM1621988, GSM1621989
NSY5	*ded1-120*	SC, 30°C to 15°C shift	(Sen et al. 2015)	GSM1621990, GSM1621991

***dom34Δ* experiment**

YNG100	*ski2Δ*	YPD, 30°C	(Guydosh and Green 2014)	GSM1279568
YNG103	*dom34Δ/ski2Δ*	YPD, 30°C	(Guydosh and Green 2014)	GSM1279569

Rli1 depletion experiment

YDY128	WT	YPD, 30°C	(Young et al. 2015)	GSM1700885
YDY130	*P_{GAL}-UBI-R-FH-RLI1*	YPD, 30°C	(Young et al. 2015)	GSM1700886, GSM1700891

***tif3Δ* SC experiment**

BY4741	WT	SC, 30°C to 15°C shift	(Sen et al. 2016)	GSM2178823, GSM2178824
FJZ052	*tif3Δ*	SC, 30°C to 15°C shift	(Sen et al. 2016)	GSM2178825, GSM2178826

Analysis of cycloheximide effects in WT cells (X1 cycloheximide and no cycloheximide data only)

BY4741	WT	YPD, 30°C	(Gerashchenko and Gladyshev 2014)	GSM1439588, GSM1439584

Ribosome profiling datasets references

Gerashchenko MV, Gladyshev VN. 2014. Translation inhibitors cause abnormalities in ribosome profiling experiments. *Nucleic Acids Res* **42**: e134.

Guydosh NR, Green R. 2014. Dom34 rescues ribosomes in 3' untranslated regions. *Cell* **156**: 950-962.

Sen ND, Zhou F, Harris MS, Ingolia NT, Hinnebusch AG. 2016. eIF4B stimulates translation of long mRNAs with structured 5' UTRs and low closed-loop potential but weak dependence on eIF4G. *Proc Natl Acad Sci U S A* **113**: 10464-10472.

Sen ND, Zhou F, Ingolia NT, Hinnebusch AG. 2015. Genome-wide analysis of translational efficiency reveals distinct but overlapping functions of yeast DEAD-box RNA helicases Ded1 and eIF4A. *Genome Res* **25**: 1196-1205.

Young DJ, Guydosh NR, Zhang F, Hinnebusch AG, Green R. 2015. Rli1/ABCE1 Recycles Terminating Ribosomes and Controls Translation Reinitiation in 3'UTRs In Vivo. *Cell* **162**: 872-884.
